# Feasibility and preliminary effects of a coping-focused dyadic family resilience intervention for breast cancer patients and their family caregivers: a pilot study

**DOI:** 10.3389/fpsyg.2026.1832777

**Published:** 2026-07-24

**Authors:** Jie Gao, Qin Shen, Pei Sun, Xiao Zhang, Hui-Ping Li, Chun-Ping Ni

**Affiliations:** 1 Department of Nursing, Air Force Medical University, Xi’An, China; 2 School of Nursing, Anhui Medical University, Hefei, China

**Keywords:** breast cancer, caregivers, coping style, family disease burden, family resilience intervention, randomized controlled trial, social support

## Abstract

**Objective:**

This pilot randomized controlled trial evaluated the feasibility and preliminary effects of a Coping-Focused Dyadic Family Resilience Intervention (CFFRI) for Chinese breast cancer survivor-caregiver dyads.

**Methods:**

A randomized, single-blind controlled trial was conducted. Sixty dyads were randomly assigned to the CFFRI group (six sessions plus usual care; *n* = 30) or the control group (usual care alone; *n* = 30). Outcomes included family resilience, coping style, family disease burden, and social support, assessed at baseline (T0), one-week post-intervention (T1), and three-month follow-up (T2). Data were analyzed using *t*-tests and generalized estimating equations (GEE). Feasibility was assessed via recruitment, retention, and adherence rates, and intervention satisfaction.

**Results:**

For the primary outcome, family resilience, the between-group mean difference at T1 was 20.08 (95% CI: 14.61–25.55) for patients and 19.85 (95% CI: 13.65–26.04) for caregivers in the per-protocol analysis, with effects sustained at T2 (patients: 14.69, 95% CI: 10.71–18.68; caregivers: 15.73, 95% CI: 11.08–20.38). Similar patterns were observed in intention-to-treat analyses. For secondary outcomes, the CFFRI group showed improvements in coping style, reduced family disease burden, and enhanced social support. Feasibility outcomes were favorable: recruitment rate 75.00%, retention rate 91.67%, high adherence rate 90.00%, and mean satisfaction score of 4.52 (SD = 0.57) out of 5.

**Conclusion:**

The CFFRI is a feasible intervention that shows promise in enhancing family resilience, improving dyadic coping, alleviating family disease burden, and strengthening social support for both patients and caregivers, with benefits sustained at 3 months. These preliminary findings provide support for its preliminary efficacy and form the basis for a future fully powered randomized controlled trial.

**Clinical trial registration:**

https://www.chictr.org.cn/showproj.html?proj=135318, identifier [ChiCTR2100052108].

## 1 Background

Breast cancer is the most frequently diagnosed malignancy and the leading cause of cancer-related death among women globally, with an annual incidence of 46.8 per 100,000 and a mortality rate of 12.7 per 100,000 (Bray et al., 2024). Its incidence continues to rise, particularly in China, where a growing number of cases are being diagnosed among younger women (Li W. et al., 2024). Concurrently, advances in cancer treatment have markedly improved survival outcomes, with breast cancer-specific survival rates now reaching 94% (Giaquinto et al., 2022). Against this backdrop of rising incidence, improved survival, and younger onset age, the quality of life of those affected has become a growing priority.

In China, socioeconomic development and increased health awareness have facilitated earlier detection, creating opportunities for timely evidence-based interventions. Despite these advances, clinical management, often involving invasive surgery and intensive treatment, can cause significant physical and psychological distress in patients (Di Nardo et al., 2022), while prolonged caregiving imposes substantial psychological morbidity and emotional burden on family caregivers (Clarijs et al., 2022). These combined challenges can disrupt family relationships, diminish intimacy, and precipitate a family crisis (Peifer et al., 2022). By contrast, some families demonstrate a capacity to adapt effectively and enact positive changes when confronting breast cancer-related challenges (Tock et al., 2025), and evidence confirms that family resilience is critical to this adaptive process (Li, 2023; Chen et al., 2024).

Nevertheless, existing research on family resilience in breast cancer has predominantly focused on identifying associated factors (Chang et al., 2022; Tao et al., 2023), whereas intervention studies remain scarce. Most available programmes are still at the pre-experimental stage (Riley et al., 2008; Saltzman, 2016), with only a few randomized controlled trials conducted to date (İnci and Temel, 2016; Wang, 2017; Yu et al., 2020). Moreover, these interventions have typically emphasized disease-related knowledge, stress management, and problem-solving skills, delivered in group formats (Weine et al., 2003; Lim and Han, 2013; İnci and Temel, 2016) and targeting patients or caregivers separately, thereby overlooking the dyadic unit as an integrated entity. Furthermore, while several dyadic interventions have been developed for chronic illness populations, most have targeted patient-spouse or parent–child dyads (McConnell et al., 2024; Giles et al., 2025; Sun et al., 2025), assuming a relatively fixed caregiver role. In breast cancer, especially among younger women, caregivers can include parents, spouses, adult children, siblings, and even close friends. To date, this heterogeneity in caregiver identity and relationship has rarely been fully addressed. Whether existing approaches are appropriate for the Chinese breast cancer population therefore remains unclear, underscoring the need for a culturally adapted dyadic intervention.

To provide a theoretical basis for such an intervention, our research group previously conducted a grounded theory study on the formation of family resilience in Chinese breast cancer families (Wu, 2020). This model identified the coping process as the core category in family resilience formation. Specifically, the coping process dynamically modulates disease perception and resilience outcomes, playing a central role in how families adjust, adapt, and overcome adversity. In parallel, Walsh’s family resilience framework conceptualizes resilience through three key processes, belief systems, organizational patterns, and communication/problem-solving (Walsh, 2003), within which coping strategies are inherently embedded. Coping is thus a modifiable process that can simultaneously strengthen the family’s belief system, enhance organizational flexibility, and improve communication. Therefore, the coping process was selected as the natural focal point for intervention.

Building on these theoretical models, we developed the Coping-Focused Dyadic Family Resilience Intervention (CFFRI) specifically for the Chinese context. The initial protocol was developed through a literature review, refined via semi-structured interviews with breast cancer dyads and clinical professionals, and subsequently revised through two rounds of expert consultation. Unlike many dyadic programmes that assume a single caregiver role, the CFFRI is adaptable to various caregiver–patient configurations, offering flexible, coping-focused strategies that can be tailored to different family structures. Moreover, the CFFRI integrates a family resilience framework with dyadic coping skills, a combination that, to our knowledge, has rarely been employed in prior interventions.

We then conducted a pilot randomized controlled trial in accordance with the SPIRIT 2013 Statement (Butcher et al., 2022) to evaluate the feasibility and preliminary effects of the CFFRI on family resilience and psychosocial well-being over 3 months. We hypothesized that, compared to standard care, the CFFRI would be associated with greater improvements in the primary outcome of family resilience at both 1 week and 3 months post-intervention. For secondary outcomes, we hypothesized potential benefits in coping ability, reduced family disease burden, and enhanced social support for both patients and caregivers. Consistent with the exploratory nature of this pilot study, these hypotheses are tentative and require confirmation in future larger-scale trials.

## 2 Methods

### 2.1 Objectives

This study aimed to evaluate the feasibility and preliminary efficacy of the Coping-Focused Dyadic Family Resilience Intervention (CFFRI) in Chinese breast cancer survivor–caregiver dyads. The primary objective was to assess its effect on enhancing family resilience. Secondary objectives were to explore its impact on coping strategies, family burden, and social support.

### 2.2 Study design

A single-blind, two-arm, parallel-group, randomized controlled trial was conducted. Given the psychosocial nature of the intervention, blinding of the interventionists was not feasible; however, outcome assessors and data analysts remained blinded to group allocation throughout the study to minimize detection bias.

### 2.3 Participants

Participants were recruited from the Breast Surgery Department of a tertiary A hospital in Hefei, Anhui Province, China, between January and October 2022. Potentially eligible patient–caregiver dyads were referred to the research team by their treating physicians. After receiving a detailed explanation of the study, those agreeing to participate provided written informed consent, with assurance that withdrawal would not affect their medical care. Inclusion criteria for breast cancer survivors were: (1) first pathological diagnosis of breast cancer; (2) age ≥ 18 years; (3) scheduled for surgical treatment; (4) smartphone user; (5) adequate communication skills and no documented history of mental disorders; and (6) willingness to provide informed consent. Inclusion criteria for family caregivers were: (1) identified as the primary caregiver (first-degree relative); (2) age ≥18 years; (3) adequate communication skills and no documented history of mental disorders; (4) smartphone user; and (5) willingness to provide informed consent. Exclusion criteria for both were: (1) presence of other severe comorbidities, and (2) concurrent participation in other psychosocial interventions.

### 2.4 Sample size

The sample size for this pilot trial was set at 30 dyads per group, based on established methodological recommendations suggesting 20–40 participants per group to adequately assess feasibility parameters (Hertzog, 2008; Sim and Lewis, 2012). With 30 dyads per group, key feasibility outcomes, such as recruitment rate (>60%), retention rate (>80%), and intervention adherence, can be estimated with acceptable precision. For example, using the Wilson method (Newcombe, 2001), a recruitment rate of 75% can be estimated with a 95% confidence interval of approximately 55 to 89%, providing a reasonable margin of error for planning a future definitive trial. Furthermore, this sample size allows for stable estimation of preliminary intervention effects and their confidence intervals, which can inform realistic power calculations and help avoid under- or over-estimation of the required sample size in a subsequent trial. The choice of 30 per group thus represents a pragmatic balance between ensuring adequate precision for feasibility estimates, managing recruitment capacity within a single hospital and study period, and minimizing participant burden.

### 2.5 Randomization and allocation

Eligible dyads providing informed consent were randomly assigned in a 1:1 ratio to either the intervention or control group. The randomization sequence was generated using SPSS (version 23.0) with a fixed random seed (20220301). Uniformly distributed random numbers (range 0–1) were generated for the planned 60 dyads, ranked, and then used to assign groups (higher rank to intervention, lower to control) in equal numbers. No blocking or stratification was applied. The final allocation sequence was documented and stored. To ensure allocation concealment, sequentially numbered, opaque, sealed envelopes were prepared by an independent researcher not involved in recruitment, intervention delivery, or outcome assessment. Each envelope contained a card indicating group assignment and was kept in a locked cabinet accessible only to that researcher. Following enrollment and baseline assessment, the next sequential envelope was opened by the same independent researcher, who then informed the intervention team of the allocation. This procedure ensured that neither the recruiting staff nor the dyads could foresee the assignment before consent and baseline measurements were completed, thereby minimizing selection bias. The two research assistants performing outcome assessments were not involved in delivering the CFFRI and had no access to the randomization list. They collected all follow-up data blinded to participants’ group assignment, and participants were instructed not to disclose their allocation. Data analyses were conducted by a statistician who received de-identified datasets with groups coded as “A” or “B”; the actual allocation was revealed only after the final analysis was completed.

### 2.6 Usual care

Both groups received usual care coordinated by the clinical unit. This routine care protocol comprised: (1) a health education handbook covering essential breast cancer information, postoperative rehabilitation exercises, lymphedema prevention guidelines, and a follow-up checklist; (2) a dedicated WeChat group used to disseminate weekly disease-related knowledge updates and provide timely responses to participants’ inquiries; and (3) facilitated peer interaction within the group to encourage sharing of positive experiences and mutual socioemotional support.

### 2.7 Intervention programme

In addition to usual care, the experimental group received the CFFRI. Grounded in the Wu family resilience process model (Wu, 2020) and Walsh’s framework (Walsh, 2003), the CFFRI was structured around three core systems and delivered through six thematic family meetings. The intervention aimed to enhance dyadic resilience by improving illness cognition, strengthening family organizational patterns and communication skills, and ultimately boosting the overall coping capacity of both patients and their primary caregivers. The CFFRI was co-delivered by a trained nursing graduate student and an oncology nurse, under the supervision of a breast cancer specialist and a psychobehavioral therapist. It consisted of six weekly online sessions, each lasting 25–30 min, a format necessitated by pandemic-related restrictions. The first session was scheduled approximately 1 week post-surgery, and all subsequent sessions were arranged in advance to accommodate each dyad’s availability. To promote participant engagement and adherence, several strategies were implemented: (1) meeting materials were distributed 1 day in advance; (2) each session began with a 2–3 min review of previous content; (3) interactive elements (e.g., videos, images, guided questions) were incorporated to encourage discussion; and (4) post-session homework was assigned to reinforce learning. A detailed outline of the CFFRI is presented in Table 1, and the full intervention protocol has been published elsewhere (Gao et al., 2022).

**Table 1:** Outline of the coping-focused dyadic family resilience intervention (CFFRI).

Item	Description
Unbreakable Belief—Know yourself and the enemy (First week after surgery)	
Theme	Understand Breast Cancer and Family Resilience
Form	Online family meeting
Target	**•** Learn about breast cancer and how it affects families. **•** Understand the concept and meaning of family resilience.
Content	**•** Ask the patient and primary caregiver to share some of their questions and concerns after patients becomes ill. **•** Explain breast cancer related knowledge to patients and their families in the form of pictures, text and videos. **•** Combine video materials and family discussions to help family members understand the characteristics of breast cancer families and help each family member understand the changes in family roles. **•** Explain the definition and importance of family resilience to patients and their families in a graphic and textual manner.
Homework	**•** Review the content of this meeting through electronic meeting materials. **•** Patients and primary caregivers share with researchers their views on breast cancer and the main effects of the disease on themselves or their families (Via voice or text message or phone call).
Unbreakable Belief—Face with smile (Second week after surgery)	
Theme	Remain Positive
Form	Online family meeting
Target	**•** Breast cancer treatment, life and psychology and other aspects care and precautions. **•** Master the basic methods to relieve stress and soothe emotions.
Content	**•** A brief recap of last week’s meeting. **•** Let the primary caregiver to review issues encountered during the patient’s care over the past week. **•** Combined with pictures and texts, it provides nursing methods and precautions for different treatments for breast cancer patients and their families. **•** Have patients and primary caregivers share their emotions and stress over the past week. **•** Help patients and primary caregivers learn ways to manage stress and emotions to stay positive and optimistic by combining pictures, text and videos.
Homework	**•** Review the content of this meeting through electronic meeting materials. **•** Patients and primary caregivers are encouraged to discuss their own stressors and ways to relieve stress with researchers (Via voice or text message or phone call).
Family Organization—Family treasure (Third week after surgery)	
Theme	Discover Available Resources
Form	Online family meeting
Target	**•** Identify the family’s own strengths and available resources and learn how to use them. **•** Summarize the experience of overcoming difficulties from past experiences.
Content	**•** A brief recap of last week’s meeting. **•** Combine pictures and words to show participants’ families what family resources, social resources and family strengths are. **•** Help family members discover and demonstrate their family strengths and resources. Discuss success and failure cases and their characteristics in overcoming difficulties through family conversations, and summarize experiences. Discuss together how to leverage existing family strengths and resources to address current issues and challenges. **•** Encourage different families to exchange experiences through WeChat groups and provide emotional support to each other.
Homework	**•** Review the content of this meeting through electronic meeting materials. **•** Give researchers examples of how their resources will be used in the future (Via voice or text message or phone call).
Family Organization—Intimacy has a way (Fourth week after surgery)	
Theme	Improve Family Cohesion
Form	Online family meeting
Target	**•** Create simple and effective family rules to promote family relationships and strengthen family bonds.
Content	**•** A brief recap of last week’s meeting. **•** Combine pictures, text and video to show patients and primary caregivers what family rules are and how they work, and encourage patients and families to share existing family rules. **•** Through family discussions, according to the specific situation of each family, clear and effective family rules are jointly formulated to enhance family interaction, strengthen family bonds, and promote family relationships.
Homework	**•** Review the content of this meeting through electronic meeting materials. **•** Patients and primary caregivers share with researchers their family relationship status, understanding and feelings about family rules (via voice or text message or phone call).
Communication and Coping—Rational communication (Fifth week after surgery)	
Theme	Facilitate Effective Communication
Form	Online family meeting
Target	• Understand what communication is and what communication problems families have. • Learn effective communication skills.
Content	• A brief recap of last week’s meeting. • Combined with video, show a case of quarrel caused by improper communication. • Combine pictures and videos to show patients and primary caregivers what communication is, and the types of family communication. • Through family discussions, help participating families identify problems in family communication and why they arise. • Introduce effective communication skills to participating families by combining pictures and videos.
Homework	• Review the content of this meeting through electronic meeting materials. • Patients and primary caregivers share a successful communication case with researchers (Via voice or text message or phone call).
Communication and Coping—Through thick and thin (Sixth week after surgery)	
Theme	Family Problems and Coping
Form	Online family meeting
Target	• Learn general ideas for problem solving. • Develop a plan to address current family issues. • Summarize the entire intervention process.
Content	• A brief recap of last week’s meeting. • Identify existing family issues through family discussions. • Combine pictures, text, and video to show patients and their families the general steps to resolve family problems. Co-exploring solutions to existing family problems following the problem-solving approach. • A brief summary of the entire intervention process.
Homework	• Review the content of this meeting through electronic meeting materials. • Patients and primary caregivers share a case of collaborative problem-solving with researchers (Via voice or text message or phone calls).

Source: Gao et al. (2022). Adapted from authors’ own work.

### 2.8 Intervention fidelity

To ensure the CFFRI was delivered as intended, several fidelity procedures were implemented. First, all facilitators received standardized training on the intervention manual and completed two simulation sessions prior to the trial. Second, with participants’ written consent, each of the six weekly sessions was audio-recorded. Third, facilitators completed a brief fidelity log after each session, confirming that all core components were delivered as outlined. Fourth, an independent research assistant randomly reviewed 20% of the audio recordings to verify adherence to the session structure and content; no major deviations were found. Weekly debriefing meetings were also held to address any implementation challenges and ensure consistency across dyads.

### 2.9 Data collection

Following eligibility screening, the study procedures were explained in detail, and written informed consent was obtained. Baseline data collection included sociodemographic characteristics, clinical profiles, and psychosocial measures encompassing family resilience, coping styles, family disease burden, and social support. Psychosocial outcomes were assessed at three time points: baseline (T0), 1 week post-intervention (T1), and three-month follow-up (T2). Feasibility metrics were evaluated upon study completion. All participants were assured of data confidentiality, with access to the final dataset restricted to the principal investigators. Data collection was conducted by two trained researchers who remained blinded to group allocation throughout the study.

### 2.10 Instruments and measures

#### 2.10.1 Baseline characteristics

Sociodemographic and clinical characteristics were collected at baseline. Sociodemographic data from both patients and caregivers included age, gender, educational level, religious affiliation, place of residence, marital status, monthly household income, medical payment method, and occupation. The specific relationship between the patient and caregiver was also documented. Clinical characteristics of the patients comprised cancer stage, surgical approach, metastasis status, and comorbidities.

#### 2.10.2 Family resilience

Family resilience was measured using the Chinese version of the Family Resilience Assessment Scale (C-FRA) (Zhang, 2022). This 28-item instrument, originally developed and validated in breast cancer populations, assesses five domains using a 5-point Likert scale. Higher total scores reflect stronger family resilience. The scale has demonstrated sound reliability and validity in Chinese populations, with a reported Cronbach’s *α* of 0.96.

#### 2.10.3 Coping style

Coping styles were assessed using the Coping Style Questionnaire (CSQ) (Lu et al., 2021), a 20-item instrument validated in breast cancer populations (Li S. et al., 2024). The scale comprises two subscales: positive coping (12 items) and negative coping (8 items), each rated from 0 to 3. The total score is calculated by subtracting the standardized negative coping score from the standardized positive coping score. A score above 0 indicates a tendency toward positive coping, while a score below 0 reflects a tendency toward negative coping. The CSQ has demonstrated good internal consistency in previous studies (Cronbach’s *α* = 0.90).

#### 2.10.4 Social support

Social support was assessed using the Social Support Scale (SSS), a 10-item instrument comprising three dimensions, which has demonstrated good reliability and validity in breast cancer populations (Liu, 2022). Total scores are categorized as follows: ≤22 (low support), 23–44 (moderate support), and 45–66 (high support). The scale has shown good internal consistency in previous validation studies (Cronbach’s *α* = 0.90).

#### 2.10.5 Family disease burden

Family disease burden was assessed using the Family Burden Scale of Disease (FBSD). This 24-item instrument comprises six dimensions, each rated from 0 to 2. Total scores are calculated by summing all items, with higher scores indicating greater family disease burden. The FBSD has demonstrated sound reliability and validity in Chinese populations (Wang et al., 2024), with good internal consistency reported in prior research (Cronbach’s *α* = 0.92).

#### 2.10.6 Feasibility assessment

Feasibility of the CFFRI was evaluated using four metrics: recruitment rate (proportion of eligible dyads who consented to participate), retention rate (proportion of consented dyads who completed all assessments), adherence (categorized as high [≥5 sessions], moderate [3–4 sessions], or low [≤2 sessions] based on session attendance), and satisfaction (measured post-intervention using a 5-point Likert scale ranging from 1 [very dissatisfied] to 5 [very satisfied]). Feasibility was assessed against four prespecified criteria: (1) recruitment rate ≥ 60%; (2) retention rate ≥ 80%; (3) high adherence (completing ≥5 of 6 sessions) ≥ 80%; and (4) mean satisfaction score ≥ 4.0. These thresholds were derived from established recommendations for pilot trial feasibility in psychosocial oncology research (Zhang et al., 2021).

### 2.11 Statistical analysis

All analyses were conducted using SPSS 23.0 (IBM, NY, USA), with statistical significance set at a two-sided *p* < 0.05. Descriptive statistics (means, standard deviations, frequencies, and percentages) were used to summarize baseline characteristics. Between-group comparisons at baseline were performed using independent *t*-tests for continuous variables and chi-square or Fisher’s exact tests for categorical variables. The primary analysis of intervention effects on family resilience and secondary outcomes was conducted using generalized estimating equations (GEE) with an autoregressive (AR1) working correlation structure, accounting for within-dyad correlations across repeated measurements (T0, T1, T2). The GEE model included group, time, and the group-by-time interaction as fixed effects, and was fitted on an intention-to-treat (ITT) basis, including all randomized dyads. The following baseline covariates were adjusted for based on *a priori* knowledge of their potential influence on psychosocial outcomes in breast cancer dyads (Lim et al., 2021; Zhang et al., 2023). For patient outcomes, the model adjusted for sociodemographic variables (age, gender, education level, religious affiliation, residence, marital status, monthly household income, medical payment method, and occupation) as well as clinical characteristics (clinical stage, surgical approach, metastasis status, and comorbidities). For caregiver outcomes, the model adjusted for the same set of sociodemographic variables, as clinical characteristics pertain specifically to patients. Adjusting for these factors helps to control for potential confounding and increases the precision of the intervention effect estimates. To account for multiple comparisons, the Bonferroni correction was applied to *post-hoc* between-group comparisons at each time point. All reported *p*-values for *post-hoc* comparisons are adjusted values. To complement the primary analysis, between-group comparisons at each post-intervention time point (T1 and T2) were also examined using independent *t*-tests. These supplementary analyses were performed on both the per-protocol (PP) population and the ITT population to examine the consistency of findings across analytic approaches. Due to the low attrition rate (5 of 60 dyads, 8.3%), missing outcome data in the ITT analyses were handled using baseline observation carried forward (BOCF). For the five dyads that dropped out during the intervention period and provided no post-baseline measurements, their baseline values were carried forward to impute missing follow-up assessments at T1 and T2. This conservative approach assumes no change from baseline for dropouts and is considered appropriate for pilot trials with minimal attrition.

## 3 Results

### 3.1 Participants

**Figure 1:**
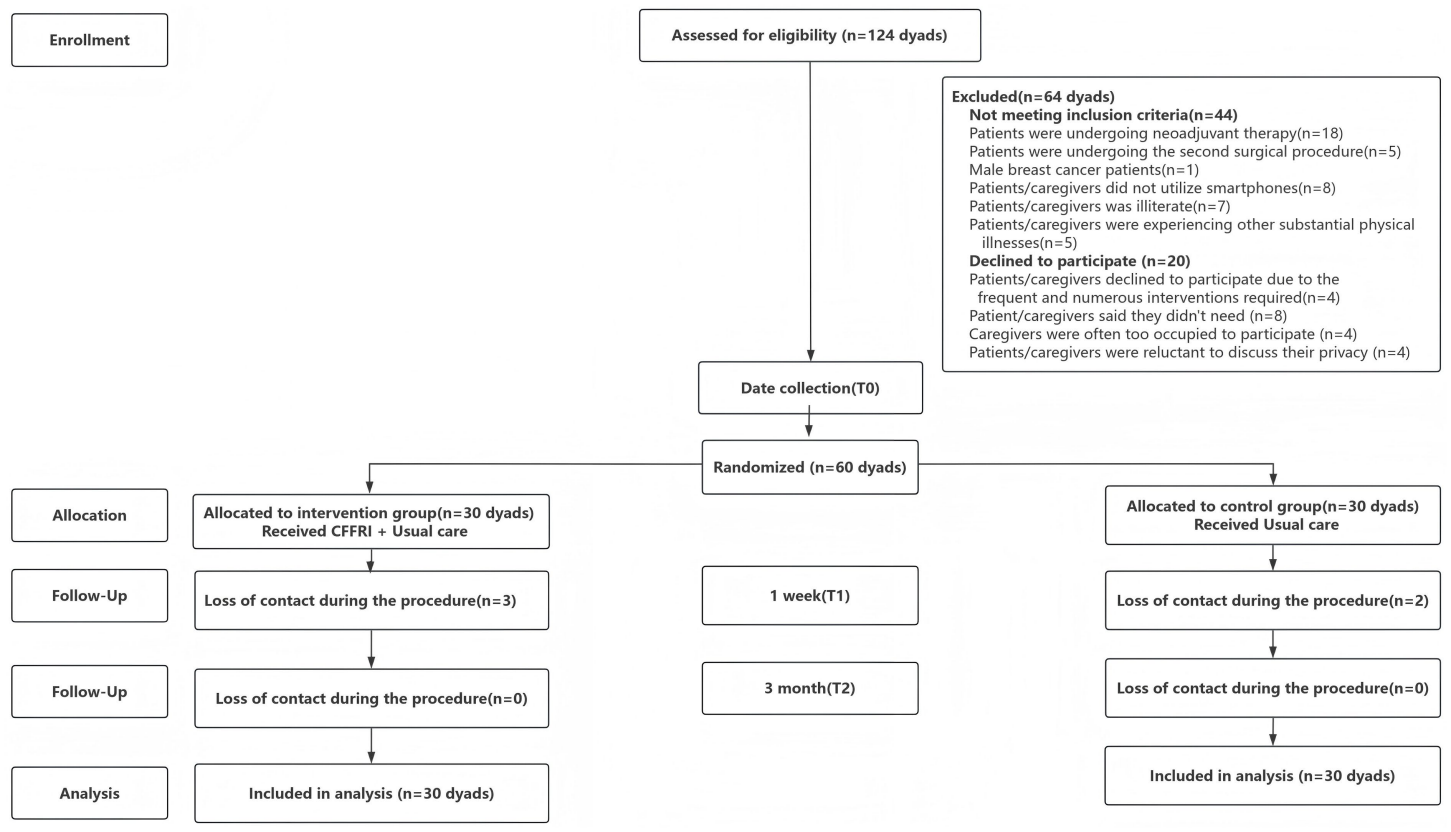
Participant CONSORT flow chart.

A total of 124 breast cancer dyads were screened for eligibility. Of these, 44 did not meet the inclusion criteria, leaving 80 eligible dyads. Among them, 20 declined to participate (perceived lack of need or privacy concerns, *n* = 12; scheduling conflicts, *n* = 4; concerns about session frequency, *n* = 4), yielding a recruitment rate of 75.00% (60 of 80 eligible dyads consented). The 60 consenting dyads were then randomized to the intervention group (*n* = 30) or the control group (*n* = 30). During the study, three dyads withdrew from the intervention group and two from the control group, giving an overall attrition rate of 8.33%. All 60 randomized dyads were included in the final analysis. The participant flow, including reasons for attrition, is presented in the CONSORT flow diagram (Figure 1). At each follow-up assessment, participants were actively asked about any discomfort or negative experiences. No adverse events or unintended consequences related to the intervention were reported throughout the study period.

### 3.2 Baseline characteristics

Baseline sociodemographic and clinical characteristics of breast cancer survivors and their family caregivers are summarized in Table 2. Among the 60 survivors (all female), the mean age was 51.47 years (SD = 11.15). Most had at least a secondary school education (66.7%), were married (95.0%), and reported no religious affiliation (88.3%). Half resided in urban areas (50.0%), and 53.3% described their financial status as fair or good. The majority were employed (85.0%) and had medical insurance (96.7%). Clinically, 56.7% were diagnosed with stage I or II disease, 81.7% had no metastasis, 65.0% underwent modified radical mastectomy, and 75.0% had no comorbidities. Among family caregivers, the mean age was 47.27 years (SD = 13.46), and 68.3% were male. Most had at least a secondary education (86.7%), were married (90.0%), and reported no religious affiliation (96.7%). Half resided in urban areas (50.0%), and 73.3% reported fair or good financial status, with 90.0% being employed. Regarding their relationship to the patient, 61.7% were spouses and 33.3% were children or parents. No statistically significant differences were observed between the intervention and control groups on any baseline sociodemographic, clinical, or outcome variables (all *p* > 0.05), indicating that randomization successfully achieved baseline comparability.

**Table 2:** Comparisons of socio-demographic and clinical characteristics and study outcomes between the intervention and control groups at baseline.

Variables	Patients *n* (%)					Caregivers *n* (%)				
	Total (*n* = 60)	Intervention (*n* = 30)	Control (*n* = 30)	*t*/*χ^2^*	*p*	Total (*n* = 60)	Intervention (*n* = 30)	Control (*n* = 30)	*t*/*χ^2^*	*p*
Age (Mean ± SD)	51.47 ± 11.15	50.90 ± 9.95	52.033 ± 12.38	−0.391	0.697	47.27 ± 13.46	44.27 ± 14.49	50.27 ± 11.84	−1.756	0.084
*Gender*									0.077	0.781
Male	0 (0.0)	0 (0.0)	0 (0.0)			41 (68.3)	21 (70.0)	20 (66.7)		
Female	60 (100.0)	30 (100.0)	30 (100.0)			19 (31.7)	9 (30.0)	10 (33.3)		
*Relationship*				2.583	0.461				2.583	0.461
Spouse	37 (61.7)	18 (60.0)	19 (63.3)			37 (61.7)	18 (60.0)	19 (63.3)		
Parent	18 (30.0)	0 (0.0)	2 (6.7)			2 (3.3)	0 (0.0)	2 (6.7)		
Children	2 (3.3)	10 (33.3)	8 (26.7)			18 (30.0)	10 (33.3)	8 (26.7)		
Sibling	3 (5.0)	2 (6.7)	1 (3.3)			3 (5.0)	2 (6.7)	1 (3.3)		
*Education level*				2.596	0.628				6.386	0.172
Primary school or less	20 (33.3)	8 (26.7)	12 (40.0)			8 (13.3)	2 (6.7)	6 (20.0)		
Secondary school	17 (28.3)	10 (33.3)	7 (23.3)			15 (25.0)	9 (30.0)	6 (20.0)		
High school	6 (10.0)	2 (6.7)	4 (13.3)			17 (28.3)	6 (20.0)	11 (36.7)		
Bachelor degree	15 (25.0)	9 (30.0)	6 (20.0)			19 (31.7)	12 (40.0)	7 (23.3)		
Master degree or above	2 (3.3)	1 (3.3)	1 (3.3)			1 (1.7)	1 (3.3)	0 (0.0)		
*Religion*				0.162	0.688				2.069	0.150
Yes	7 (11.7)	3 (10.0)	4 (13.3)			2 (3.3)	0 (0.0)	2 (6.7)		
No	53 (88.3)	27 (90.0)	26 (86.7)			58 (96.7)	30 (100.0)	28 (93.3)		
*Residence*				4.600	0.204				4.600	0.204
Rural area	20 (33.3)	7 (23.3)	13 (43.3)			20 (33.3)	7 (23.3)	13 (43.3)		
Town	4 (6.7)	2 (6.7)	2 (6.7)			4 (6.7)	2 (6.7)	2 (6.7)		
County	6 (10.0)	5 (16.7)	1 (3.3)			6 (10.0)	5 (16.7)	1 (3.3)		
Urban area	30 (50.0)	16 (53.3)	14 (46.7)			30 (50)	16 (53.3)	14 (46.7)		
*Marital status*				3.158	0.368				2.963	0.085
Unmarried	2 (3.33)	0 (0.0)	2 (6.7)			6 (10.0)	5 (16.7)	1 (3.3)		
Married	57 (95.0)	30 (100)	27 (90.0)			54 (90.0)	25 (83.3)	29 (96.7)		
Widowed	1 (1.7)	0 (0.0)	1 (3.3)			0 (0.0)	0 (0.0)	0 (0.0)		
*Monthly household income (Yuan)*				4.982	0.173				5.733	0.125
<1,000	11 (18.3)	5 (16.7)	6 (20.0)			6 (10.0)	5 (16.7)	1 (3.3)		
1,000~	17 (28.3)	5 (16.7)	12 (40.0)			10 (16.7)	3 (10.0)	7 (23.3)		
3,000~	21 (35.0)	13 (43.3)	8 (26.7)			20 (33.3)	8 (26.7)	12 (40)		
≥5,000	11 (18.3)	7 (23.3)	4 (13.3)			24 (40.0)	14 (46.7)	10 (33.3)		
*Payment methods*				2.069	0.355				4.000	0.135
Self-paying	1 (1.7)	0 (0.0)	1 (3.3)			2 (3.3)	2 (6.7)	0 (0.0)		
Commercial insurance	1 (1.7)	0 (0.0)	1 (3.3)			2 (3.3)	0 (0.0)	2 (6.7)		
Medical insurance	58 (96.7)	30 (100)	28 (93.3)			56 (93.3)	28 (93.3)	28 (93.3)		
*Occupation*				9.600	0.294				13.998	0.082
Unemployed	9 (15.0)	3 (10.0)	6 (20.0)			6 (10.0)	3 (10.0)	3 (10.0)		
Worker	5 (8.3)	2 (6.7)	3 (10.0)			8 (13.3)	4 (13.3)	4 (13.3)		
Farmer	15 (25.0)	6 (20.0)	9 (30.0)			5 (8.3)	1 (3.3)	4 (13.3)		
Businessman	3 (5.0)	3 (10.0)	0 (0.0)			5 (8.3)	0 (0.0)	5 (16.7)		
Services industry	4 (6.7)	3 (10.0)	1 (3.3)			3 (5.0)	2 (6.7)	1 (3.3)		
Teacher	6 (10.0)	4 (13.3)	2 (6.7)			4 (6.7)	2 (6.7)	2 (6.7)		
Health worker	1 (1.7)	0 (0.0)	1 (3.3)			3 (5.0)	0 (0.0)	3 (10.0)		
Government official	5 (8.3)	4 (13.3)	1 (3.3)			7 (11.7)	5 (16.7)	2 (6.7)		
Others	12 (20.0)	5 (16.7)	7 (23.3)			19 (31.7)	13 (43.3)	6 (20.0)		
*Clinical stage*				0.278	0.870					
I	13 (21.7)	6 (20.0)	7 (23.3)							
II	21 (35.0)	10 (33.3)	11 (36.7)							
III	26 (43.3)	14 (46.7)	12 (40.0)							
*Surgical approaches*				3.675	0.299					
Radical mastectomy	9 (15.0)	2 (6.7)	7 (23.3)							
Modified radical mastectomy	39 (65.0)	21 (70.0)	18 (60.0)							
Conserving surgery	6 (10.0)	3 (10.0)	3 (10.0)							
Simple mastectomy	6 (10.0)	4 (13.3)	2 (6.7)							
*Metastasis*				1.002	0.317					
Yes	11 (18.3)	4 (13.3)	7 (23.3)							
No	49 (81.7)	26 (86.7)	23 (76.7)							
*Comorbidities*				2.222	0.136					
Yes	15 (25.0)	5 (16.7)	10 (33.3)							
No	45 (75.0)	25 (83.3)	20 (66.7)							
Family resilience (RR)		97.83 ± 10.82	102.17 ± 16.01	−1.228	0.224		99.7 ± 9.72	103.23 ± 18.03	−0.945	0.349
Coping style (CS)		0.01 ± 0.34	0.18 ± 0.58	−1.314	0.194		0.16 ± 0.44	0.2 ± 0.59	−0.302	0.764
Family disease burden (FDB)		12.67 ± 5.86	11.1 ± 5.21	1.095	0.278		12.47 ± 5.24	12.2 ± 6.05	0.182	0.856
Social support (SS)		43.67 ± 6.71	43.5 ± 6.6	0.097	0.923		44.27 ± 5.83	43.47 ± 6.83	0.488	0.627

### 3.3 Patients’ outcomes

**Figure 2:**
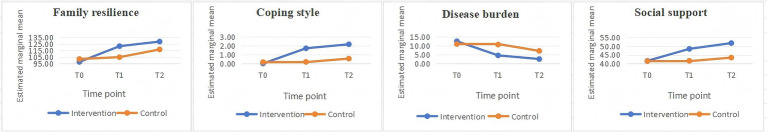
Profile plots of patients.

Patient outcomes across the three time points are summarized in Tables 3, 4. Figure 2 illustrates temporal changes in estimated marginal means for family resilience, coping style, family disease burden, and social support. Primary outcome. For family resilience, GEE analysis revealed a significant time-by-group interaction effect (*p* < 0.001; Table 3). *Post-hoc* comparisons indicated that patients in the CFFRI group reported significantly higher family resilience than those in the control group at both T1 and T2 (all *p* < 0.01; Table 4). In the per-protocol analysis, the mean difference was 20.08 (95% CI: 14.61–25.55) at T1 and 14.69 (95% CI: 10.71–18.68) at T2. Similar patterns were observed in the intention-to-treat analysis (T1: 16.60, 95% CI: 10.78–22.42; T2: 11.93, 95% CI: 6.32–17.55). Secondary outcomes. For coping style, family disease burden, and social support, GEE analyses also revealed significant time-by-group interaction effects (all *p* < 0.001; Table 3). Post-hoc comparisons showed that, compared to the control group, patients in the CFFRI group demonstrated significantly more positive coping styles, lower family disease burden, and higher social support at both T1 and T2 (all *p* < 0.001; Table 4). Mean differences and 95% CIs for these outcomes are detailed in Table 4. These improvements were consistently supported in both per-protocol and intention-to-treat analyses.

**Table 3:** Testing the effects of generalized estimation equation models on participants’ family resilience, coping style, family disease burden, and social support level.

Variable	Patients			Caregivers		
	Group	Time	Group × Time	Group	Time	Group × Time
	Wald *χ*^2^	Wald *χ*^2^	Wald *χ*^2^	Wald *χ*^2^	Wald *χ*^2^	Wald *χ*^2^
Family resilience (FR)	10.476^⁎⁎^	175.297^⁎⁎⁎^	155.818^⁎⁎⁎^	12.886^⁎⁎⁎^	160.871^⁎⁎⁎^	138.812^⁎⁎⁎^
Positive outlook	11.710^⁎⁎^	152.976^⁎⁎⁎^	93.979^⁎⁎⁎^	16.708^⁎⁎⁎^	115.263^⁎⁎⁎^	86.288^⁎⁎⁎^
Family contectedness	24.373^⁎⁎⁎^	150.482^⁎⁎⁎^	187.780^⁎⁎⁎^	26.628^⁎⁎⁎^	130.574^⁎⁎⁎^	181.129^⁎⁎⁎^
Social resources	4.247^⁎^	102.500^⁎⁎⁎^	97.290^⁎⁎⁎^	6.141^⁎^	106.695^⁎⁎⁎^	76.052^⁎⁎⁎^
Clarity of communication	3.823	109.567^⁎⁎⁎^	33.351^⁎⁎⁎^	5.032^⁎^	84.937^⁎⁎⁎^	28.532^⁎⁎⁎^
Collaborative problem-solving	2.915	108.824^⁎⁎⁎^	26.941^⁎⁎⁎^	1.981	99.649^⁎⁎⁎^	31.782^⁎⁎⁎^
Coping style (CS)	75.566^⁎⁎⁎^	222.515^⁎⁎⁎^	201.338^⁎⁎⁎^	76.554^⁎⁎⁎^	207.092^⁎⁎⁎^	198.367^⁎⁎⁎^
Family disease burden (FBD)	10.582^⁎⁎^	95.391^⁎⁎⁎^	43.600^⁎⁎⁎^	8.756^⁎⁎^	142.023^⁎⁎⁎^	96.062^⁎⁎⁎^
Social support (SS)	17.780^⁎⁎⁎^	84.701^⁎⁎⁎^	72.409^⁎⁎⁎^	7.601^⁎⁎^	141.073^⁎⁎⁎^	74.145^⁎⁎⁎^

^⁎^*p* < 0.05.

^⁎⁎^*p* < 0.01.

^⁎⁎⁎^*p* < 0.001.

**Table 4:** Comparison of participants’ family resilience, coping style, family disease burden, and social support level between two groups for per-protocol and intention-to-treat analyses.

Variable	Time	Patients				Caregivers			
		CG (M ± SD)	IG (M ± SD)	Mean difference (95% CI)	*t*	CG (M ± SD)	IG (M ± SD)	Mean difference (95% CI)	*t*
Per protocol (IG = 27 dyads, CG = 28 dyads)									
Family resilience (FR)	T1	104.50 ± 12.36	124.58 ± 6.05	20.08 (14.61, 25.55)	7.439^⁎⁎⁎^	105.46 ± 14.18	125.31 ± 6.39	19.85 (13.65, 26.04)	6.507^⁎⁎⁎^
	T2	117.92 ± 9.55	132.62 ± 2.74	14.69 (10.71, 18.68)	7.537^⁎⁎⁎^	117.50 ± 11.30	133.23 ± 2.41	15.73 (11.08, 20.38)	6.943^⁎⁎⁎^
Positive outlook	T1	26.31 ± 3.11	31.65 ± 1.65	5.35 (3.96, 6.73)	7.749^⁎⁎⁎^	26.54 ± 3.90	31.92 ± 2.00	5.39 (3.64, 7.13)	6.264^⁎⁎⁎^
	T2	29.81 ± 2.76	33.54 ± 0.81	3.73 (2.60, 4.88)	6.619^⁎⁎⁎^	29.54 ± 3.22	33.85 ± 0.78	4.31 (2.98, 5.64)	6.637^⁎⁎⁎^
Family contectedness	T1	31.04 ± 3.50	37.85 ± 1.83	6.81 (5.24, 8.38)	8.785^⁎⁎⁎^	31.19 ± 3.82	37.81 ± 1.74	6.62 (4.95, 8.29)	8.040^⁎⁎⁎^
	T2	34.08 ± 3.17	39.12 ± 0.95	5.04 (3.71, 6.37)	7.753^⁎⁎⁎^	34.04 ± 3.38	39.27 ± 1.04	5.23 (3.82, 6.65)	7.549^⁎⁎⁎^
Social resources	T1	18.58 ± 2.08	22.35 ± 1.55	3.77 (2.75, 4.79)	7.409^⁎⁎⁎^	18.88 ± 2.52	22.38 ± 1.50	3.5 (2.34, 4.67)	6.088^⁎⁎⁎^
	T2	21.08 ± 1.74	23.62 ± 0.80	2.54 (1.78, 3.30)	6.747^⁎⁎⁎^	20.85 ± 2.29	23.65 ± 1.02	2.81 (1.81, 3.81)	5.708^⁎⁎⁎^
Clarity of communication	T1	14.31 ± 2.46	16.58 ± 1.36	2.27 (1.15, 3.39)	4.113^⁎⁎⁎^	14.50 ± 2.66	16.77 ± 1.21	2.27 (1.11, 3.43)	3.963^⁎⁎⁎^
	T2	16.46 ± 1.58	18.54 ± 0.86	2.08 (1.36, 2.79)	5.886^⁎⁎⁎^	16.69 ± 1.67	18.54 ± 0.65	1.85 (1.13, 2.56)	5.262^⁎⁎⁎^
Collaborative problem-solving	T1	14.27 ± 2.33	16.15 ± 1.38	1.89 (0.81, 2.96)	3.557^⁎⁎^	14.35 ± 2.36	16.42 ± 1.33	2.08 (1.01, 3.15)	3.901^⁎⁎⁎^
	T2	16.50 ± 1.45	17.81 ± 0.80	1.31 (0.65, 1.97)	4.027^⁎⁎⁎^	16.38 ± 1.77	17.92 ± 0.98	1.54 (0.74, 2.34)	3.884^⁎⁎⁎^
Coping style (CS)	T1	0.17 ± 0.48	1.92 ± 0.30	1.75 (1.53, 1.98)	15.822^⁎⁎⁎^	0.15 ± 0.42	2.01 ± 0.25	1.85 (1.66, 2.05)	19.476^⁎⁎⁎^
	T2	0.60 ± 0.65	2.44 ± 0.23	1.85 (1.57, 2.12)	13.641^⁎⁎⁎^	0.51 ± 0.59	2.48 ± 0.29	1.97 (1.71, 2.23)	15.246^⁎⁎⁎^
Family disease burden (FBD)	T1	11.15 ± 6.37	3.73 ± 2.63	−7.42 (−10.17, −4.67)	−5.490^⁎⁎⁎^	11.38 ± 4.71	3.65 ± 2.13	−7.73 (−9.79, −5.67)	−7.626^⁎⁎⁎^
	T2	7.00 ± 3.25	1.35 ± 1.16	−5.65 (−7.03, −4.27)	−8.352^⁎⁎⁎^	4.88 ± 3.20	1.46 ± 1.39	−3.42 (−4.80, −2.05)	−4.996^⁎⁎⁎^
Social support (SS)	T1	41.31 ± 6.46	49.27 ± 4.45	7.96 (4.87, 11.05)	5.175^⁎⁎⁎^	41.96 ± 6.37	49.08 ± 4.94	7.12 (3.94, 10.29)	4.500^⁎⁎⁎^
	T2	43.38 ± 6.18	53.04 ± 3.47	9.65 (6.84, 12.47)	6.942^⁎⁎⁎^	48.73 ± 5.55	52.96 ± 4.06	4.23 (1.52, 6.94)	3.135^⁎⁎^
Intention-to-treat (IG = 30 dyads, CG = 30 dyads)									
Family resilience (FR)	T1	105.00 ± 11.88	121.60 ± 10.62	16.60 (10.78, 22.42)	5.706^⁎⁎⁎^	105.97 ± 13.41	122.50 ± 10.08	16.53 (10.40, 22.66)	5.399^⁎⁎⁎^
	T2	116.63 ± 9.88	128.57 ± 11.76	11.93 (6.32, 17.55)	4.255^⁎⁎⁎^	116.40 ± 11.09	129.37 ± 10.90	12.97 (7.29, 18.65)	4.568^⁎⁎⁎^
Positive outlook	T1	26.37 ± 2.94	30.80 ± 3.15	4.43 (2.86, 6.01)	5.639^⁎⁎⁎^	26.60 ± 3.66	31.17 ± 2.83	4.57 (2.88, 6.26)	5.404^⁎⁎⁎^
	T2	29.40 ± 2.82	32.43 ± 3.38	3.03 (1.42, 4.64)	3.772^⁎⁎⁎^	29.20 ± 3.16	32.83 ± 2.85	3.63 (2.08, 5.19)	4.677^⁎⁎⁎^
Family contectedness	T1	31.20 ± 3.31	37.03 ± 2.79	5.83 (4.25, 7.41)	7.392^⁎⁎⁎^	31.27 ± 3.55	37.00 ± 2.77	5.73 (4.09, 7.38)	6.975^⁎⁎⁎^
	T2	33.83 ± 3.04	38.13 ± 2.78	4.3 (2.80, 5.81)	5.720^⁎⁎⁎^	33.73 ± 3.24	38.27 ± 2.89	4.53 (2.95, 6.12)	5.724^⁎⁎⁎^
Social resources	T1	18.73 ± 2.05	21.67 ± 2.54	2.93 (1.74, 4.13)	4.926^⁎⁎⁎^	18.93 ± 2.38	21.73 ± 2.49	2.8 (1.54, 4.06)	4.455^⁎⁎⁎^
	T2	20.90 ± 1.77	22.77 ± 2.58	1.87 (0.72, 3.01)	3.267^⁎⁎^	20.63 ± 2.24	22.83 ± 2.61	2.2 (0.94, 3.46)	3.503^⁎⁎^
Clarity of communication	T1	14.47 ± 2.53	16.20 ± 1.75	1.73 (0.61, 2.86)	3.087^⁎⁎^	14.67 ± 2.63	16.50 ± 1.41	1.83 (0.74, 2.93)	3.366^⁎⁎^
	T2	16.33 ± 1.81	17.90 ± 1.97	1.57 (0.59, 2.54)	3.209^⁎⁎^	16.57 ± 1.78	18.03 ± 1.52	1.47 (0.61, 2.32)	3.438^⁎⁎^
Collaborative problem-solving	T1	14.23 ± 2.21	15.90 ± 1.58	1.67 (0.67, 2.66)	3.360^⁎⁎^	14.50 ± 2.30	16.10 ± 1.58	1.6 (0.58, 2.62)	3.138^⁎⁎^
	T2	16.17 ± 1.66	17.33 ± 1.58	1.17 (0.33, 2.01)	2.784^⁎⁎^	16.27 ± 1.76	17.40 ± 1.71	1.13 (0.24, 2.03)	2.526^⁎^
Coping style (CS)	T1	0.20 ± 0.51	1.74 ± 0.62	1.54 (1.25, 1.83)	10.549^⁎⁎⁎^	0.21 ± 0.50	1.82 ± 0.64	1.61 (1.31, 1.90)	10.815^⁎⁎⁎^
	T2	0.57 ± 0.65	2.19 ± 0.74	1.62 (1.26, 1.98)	8.979^⁎⁎⁎^	0.53 ± 0.61	2.23 ± 0.79	1.70 (1.34, 2.07)	9.354^⁎⁎⁎^
Family disease burden (FBD)	T1	10.77 ± 6.07	4.63 ± 3.72	−6.13 (−8.74, −3.53)	−4.718^⁎⁎⁎^	10.70 ± 4.86	4.50 ± 3.42	−6.2 (−8.37, −4.03)	−5.715^⁎⁎⁎^
	T2	7.17 ± 3.19	2.57 ± 3.68	−4.6 (−6.38, −2.82)	−5.175^⁎⁎⁎^	5.07 ± 3.23	2.60 ± 3.65	−2.47 (−4.25, −0.69)	−2.772^⁎⁎⁎^
Social support (SS)	T1	41.70 ± 6.23	48.47 ± 5.16	6.77 (3.81, 9.73)	4.579^⁎⁎⁎^	42.50 ± 6.12	48.37 ± 5.64	5.87 (2.83, 8.91)	3.861^⁎⁎⁎^
	T2	43.50 ± 5.91	51.73 ± 5.21	8.23 (5.36, 11.11)	5.728^⁎⁎⁎^	48.37 ± 5.29	51.73 ± 5.64	1.41 (0.54, 6.19)	2.386^⁎⁎⁎^

1 week (T1), 3 months (T2) post intervention; mean (M), standard deviation (SD); Intervention group (IG), control group (CG).

^⁎^*p* < 0.05.

^⁎⁎^*p* < 0.01.

^⁎⁎⁎^*p* < 0.001.

### 3.4 Family caregivers’ outcomes

**Figure 3:**
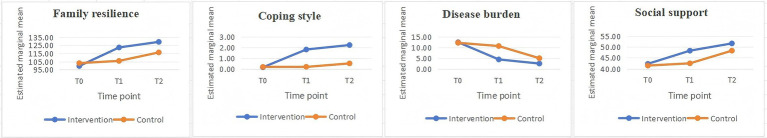
Profile plots of caregivers.

Outcomes for family caregivers across the three time points are summarized in Tables 3, 4. Figure 3 depicts temporal changes in estimated marginal means for family resilience, coping style, family disease burden, and social support. Primary outcome. For family resilience, GEE analysis revealed a significant time-by-group interaction effect (*p* < 0.001; Table 3). *Post-hoc* comparisons indicated that caregivers in the CFFRI group reported significantly higher family resilience than those in the control group at both T1 and T2 (all *p* < 0.05; Table 4). In the per-protocol analysis, the mean difference was 19.85 (95% CI: 13.65–26.04) at T1 and 15.73 (95% CI: 11.08–20.38) at T2. Similar patterns were observed in the intention-to-treat analysis (T1: 16.53, 95% CI: 10.40–22.66; T2: 12.97, 95% CI: 7.29–18.65). Secondary outcomes. For coping style, family disease burden, and social support, GEE analyses also revealed significant time-by-group interaction effects (all *p* < 0.001; Table 3). Post-hoc comparisons showed that, compared to the control group, caregivers in the CFFRI group demonstrated significantly more effective coping styles, lower family disease burden, and greater social support at both T1 and T2 (all *p* < 0.01; Table 4). Mean differences and 95% CIs for these outcomes are detailed in Table 4. These findings were consistent across both per-protocol and intention-to-treat analyses.

### 3.5 Feasibility and satisfaction

All four prespecified feasibility criteria were met or exceeded. The recruitment rate was 75% (60 of 80 eligible dyads consented), surpassing the ≥60% threshold. The retention rate was 91.67% (55 of 60 dyads completed all follow-up assessments), exceeding the ≥80% criterion. Regarding intervention adherence, 90.00% (27/30) of dyads were highly adherent (completing ≥5 of 6 sessions), 3.33% (1/30) were moderately adherent (3–4 sessions), and 6.67% (2/30) showed low adherence (≤2 sessions), with the high adherence rate meeting the ≥80% benchmark. The mean satisfaction score was 4.52 (SD = 0.57) among the 27 dyads who completed the post-intervention survey, exceeding the ≥4.0 threshold. These findings indicate that the CFFRI was feasible and well-accepted in this pilot context.

## 4 Discussion

This pilot randomized controlled trial represents the first implementation and evaluation of the Coping-Focused Dyadic Family Resilience Intervention (CFFRI), a program specifically developed for Chinese breast cancer patient–caregiver dyads. The CFFRI shifts the intervention focus from the individual to the dyadic level, aiming to systematically strengthen family resilience through enhanced coping strategies. The results suggest that the intervention was associated with significant improvements in family resilience and coping capacity, along with increased social support and reduced family disease burden among both patients and caregivers. These findings offer a practical framework for improving postoperative home care management in this population.

### 4.1 Effects of the CFFRI on family resilience and coping style

As a chronic condition with a high survival rate, breast cancer involves prolonged treatment and recovery that continually challenge the psychological and physical well-being of both patients and their family caregivers. These sustained demands may gradually erode dyadic resilience and adaptive coping over time (Chang et al., 2022; Shi et al., 2023), underscoring the need for targeted interventions that strengthen resilience and crisis response capabilities. While existing approaches have typically focused on patients or caregivers individually (Lim and Han, 2013; İnci and Temel, 2016), the CFFRI adopts a dyadic perspective that addresses the shared illness experience within the caregiver–patient unit. By integrating coping skills training into a family resilience framework, the intervention seeks to enhance core relational processes, such as communication, mutual support, and shared meaning-making, thereby helping dyads collectively navigate health-related challenges.

Consistent with its theoretical underpinnings, the CFFRI was associated with substantial improvements in overall family resilience and most of its specific domains among breast cancer patients compared to the control group, with these gains maintained throughout the three-month follow-up. Generalized estimating equation (GEE) analyses revealed significant time-by-group interaction effects for both comprehensive family resilience and the majority of its constituent dimensions, a pattern consistently observed within the caregiver cohort. These findings align with earlier evidence that theory-driven interventions can improve family resilience in chronic illness (Murta et al., 2021). Of particular note were the more limited effects observed in the collaborative problem-solving domain. This pattern may be attributable to the intervention’s relatively condensed timeframe; the development of shared problem-solving capacities typically requires extended practice and contextual application. Future refinements to the program might therefore incorporate more extensive, scenario-based exercises and extended skill-building opportunities to strengthen this dimension.

To better understand how these improvements may have been achieved, we explored the potential mechanisms through which the CFFRI could influence family resilience and coping. First, by explicitly targeting coping as the core mechanism of change, as derived from Wu’s family resilience process model (Wu, 2020), the intervention equipped dyads with concrete skills to reframe illness-related appraisals and to collaboratively address challenges. This shift from passive adaptation to active problem-solving may have reinforced the family’s belief system and its capacity to find meaning in adversity, consistent with Walsh’s framework (Walsh, 2003). Second, the dyadic format likely facilitated shared emotional processing and mutual support, which may have reinforced family connectedness and clarified communication, both key components of resilience. Third, the structured yet flexible nature of the sessions allowed dyads to practice coping strategies in real time and receive immediate feedback, which may have promoted self-efficacy and reduced feelings of helplessness. These mechanisms are supported by qualitative participant feedback indicating strengthened interpersonal relationships and increased confidence in managing health-related challenges.

Correspondingly, participants in the CFFRI group showed enhanced coping capabilities relative to the control condition. GEE modeling revealed significant main effects and time-by-group interactions, suggesting the program’s value in fostering adaptive dyadic coping through its integrated focus on knowledge acquisition, emotional regulation, and collaborative strategy development. These results align with previous work by Wang (2017), further reinforcing the value of coping-focused interventions in chronic illness populations.

Beyond statistical significance, the magnitude of the observed improvements supports their practical relevance. The gains in family resilience exceeded the minimal clinically important difference reported in prior research (İnci and Temel, 2016), suggesting that the intervention produced changes likely to be perceptible in dyads’ daily functioning. Notably, these benefits were sustained over the three-month follow-up period. The shift in coping style was equally pronounced, reflecting a meaningful transition from passive adaptation toward more active and collaborative strategies. The durability of these changes suggests that the coping skills acquired during the intervention were successfully integrated into dyads’ everyday lives, contributing to lasting improvements in how families manage cancer-related challenges.

To further contextualize our findings within the broader literature, we compared the CFFRI with previously published dyadic and family-based interventions. The magnitude and pattern of the CFFRI effects are broadly consistent with those reported in earlier family resilience interventions. For instance, theory-driven programs based on McCubbin’s framework have shown improvements in family resilience among families affected by schizophrenia (Lim and Han, 2013). Similarly, a support program for stroke families reported enhanced resilience and coping (İnci and Temel, 2016), and an empowerment-based intervention for families of children with leukemia also demonstrated improved family functioning (Wang, 2017). Collectively, these findings, together with ours, support the general effectiveness of theory-driven family resilience interventions across diverse chronic illness populations.

However, the CFFRI differs from most prior interventions in several important respects. First, whereas the majority of published family resilience interventions employ multi-family face-to-face group sessions (Lim and Han, 2013; İnci and Temel, 2016), the CFFRI adopts a dyadic online format. This distinction is particularly relevant in the Chinese cultural context, where family illness is often regarded as a private matter; multi-family meetings may raise privacy concerns and limit scheduling flexibility, while the dyadic online format offers greater accessibility and convenience. Second, most existing sessions last 30–90 min (Wang, 2017; Yu et al., 2020) whereas the CFFRI sessions are 25–30 min. This shorter duration may reduce participant burden while maintaining effectiveness, an important consideration for families managing the multifaceted demands of cancer care. Third, most prior interventions assume a fixed caregiver role (typically parents or spouse) (Chartier et al., 2010), whereas breast cancer involves diverse caregivers, including spouses, parents, adult children, and siblings. The CFFRI was specifically designed to accommodate this heterogeneity, making it more adaptable to real-world family configurations. These distinctive features may explain why the CFFRI achieved consistent effects across diverse dyad types, regardless of the caregiver–patient relationship. Nevertheless, direct comparisons of effect sizes remain challenging due to variations in outcome measures and follow-up durations across studies. Future research employing standardized assessments would facilitate more precise cross-study comparisons and further clarify the relative effectiveness of different intervention formats and durations.

### 4.2 Effects of the CFFRI on family disease burden and social support

Dyads affected by breast cancer often experience notable challenges across physical, psychological, and social domains (Ellis et al., 2021; Roberson et al., 2022). Patients may struggle with postoperative pain, body image changes, and reduced social participation, all of which contribute to the perceived burden of the disease (Di Nardo et al., 2022). Caregivers, in turn, face physical exhaustion, emotional strain, and role-related conflicts resulting from sustained care responsibilities (Clarijs et al., 2022). Additionally, the protracted nature of breast cancer treatment may present economic concerns for many families (Erfani et al., 2021). In this context, therapeutic education and problem-solving training have been shown to help alleviate the overall disease burden for dyads managing chronic illness (Mou et al., 2023). Consistent with this, in the present study, both patients and caregivers in the CFFRI group reported significantly lower family disease burden at both post-intervention assessments compared to the control group. GEE analyses further revealed a significant time-by-group interaction effect on family disease burden across both patient and caregiver reports.

Similarly, social support, an essential resource for dyads coping with cancer, is often compromised throughout the illness trajectory (Jadidi and Ameri, 2022). Diminished social connectedness can exacerbate perceived burden and limit opportunities for meaningful engagement. In this trial, participants in the CFFRI group showed significantly higher levels of social support at both follow-up assessments relative to the control group. GEE models also revealed a significant time-by-group interaction for social support in both patients and caregivers, suggesting that the intervention may have enhanced dyads’ ability to identify, access, and utilize relational and community resources. These results align with earlier work by Weine et al. (2003) and İnci and Temel (2016), and reinforce the value of coping-focused resilience interventions in strengthening social connectedness and resource mobilization among breast cancer dyads.

Several mechanisms may account for these improvements. The intervention’s emphasis on structured problem-solving and dyadic communication likely equipped families with more effective strategies to manage daily care demands, thereby alleviating both practical and emotional caregiving strain. In addition, the normalization of distress through psychoeducation may have reduced feelings of isolation and guilt, common contributors to perceived burden. The concurrent enhancement of social support may reflect the intervention’s success in helping dyads identify and mobilize existing relational and community resources, a process facilitated by peer interaction and the emphasis on shared coping. By fostering a sense of shared responsibility and mutual reliance, the CFFRI may have counteracted the social withdrawal often observed in cancer dyads, thereby strengthening both perceived and received support networks.

The magnitude of the observed changes underscores their clinical relevance. The reduction in family disease burden was substantial and sustained, likely reflecting meaningful alleviation of the practical and emotional strain that families experience during cancer caregiving. The improvements in social support were similarly pronounced, suggesting a meaningful strengthening of relational and community resources that may buffer the psychosocial impact of cancer. These consistent and sizable changes indicate that the intervention’s benefits extend beyond resilience to tangible aspects of dyadic well-being.

### 4.3 Feasibility of the CFFRI in breast cancer dyads

Feasibility assessment is a critical step in developing complex behavioral interventions for vulnerable populations (Zhang et al., 2021). In this study, all four prespecified feasibility criteria were met or exceeded, yielding valuable insights for future implementation. The recruitment rate of 75% compares favorably with rates reported in similar psychosocial oncology trials (Custers et al., 2023). Among eligible dyads who declined, the most common reason was perceived lack of need, suggesting that families with lower distress or higher baseline functioning may not view such interventions as relevant. This highlights the importance of screening tools to identify families most likely to benefit, or offering modular options tailored to specific needs. Work-related time constraints, cited by 20% of those who declined, underscore the value of the flexible online format adopted in this trial. The retention rate of 91.7% is comparable to or higher than rates in other dyadic intervention studies (Mou et al., 2023). The timing of attrition, two dyads lost contact early, suggests that initial engagement represents a critical window. One dyad’s withdrawal after four sessions due to perceived normalization of family functioning indicates that for some families, benefits may be achieved earlier than anticipated. Intervention adherence was high, with 90% of dyads completing at least five of six sessions. The small proportion with low adherence likely reflects competing work or caregiving demands, pointing to the need for flexible scheduling in future implementations. Satisfaction was universal among those who completed the survey, likely attributable to the intervention’s dyadic, family-centered design, the convenience of online delivery, and the culturally sensitive adaptation of content to the Chinese context.

These feasibility findings carry several implications for scale-up. The high retention and adherence rates, together with universal satisfaction, support the acceptability of the CFFRI in this population. The recruitment patterns suggest that future trials would benefit from targeted recruitment and pre-enrollment screening. Offering evening or weekend sessions could address work-related barriers, and modularized content may accommodate families with varying needs. The universal satisfaction with online delivery supports broader dissemination through telehealth platforms. Collectively, these outcomes support the conduct of a future fully powered randomized controlled trial, with refinements to recruitment and adherence strategies as outlined.

### 4.4 Cultural and methodological considerations

The delivery and effects of the CFFRI should be understood within the Chinese cultural context. Several cultural factors may have influenced both the implementation and outcomes of the intervention. First, the dyadic format aligns well with the traditional Chinese family structure, which emphasizes interdependence, filial piety, and collective coping in the face of illness (Lu, 2009). In Chinese culture, family members are expected to share responsibility for caregiving and decision-making, making a dyadic approach more ecologically valid than interventions targeting patients or caregivers individually. This cultural congruence may partly explain the high retention and adherence rates. Second, the online delivery format proved particularly well-suited to the Chinese context, where digital platforms such as WeChat are widely embedded in daily communication and family coordination. This format may have enhanced accessibility for families with geographically separated members, while also addressing privacy concerns associated with multi-family face-to-face sessions. Third, the diversity of caregiver configurations in this study, including spouses, parents, adult children, and siblings, reflects the reality of Chinese caregiving networks. The CFFRI’s adaptability to various caregiver–patient configurations may therefore be particularly relevant to Chinese family dynamics, where caregiving roles are flexible and distributed among family members. However, these cultural factors also have implications for replicability. In settings where individualism is more emphasized or family structures are more nuclear, the effectiveness of a dyadic, coping-focused intervention may differ. Future replication studies in other cultural settings should consider modifications to delivery format, caregiver inclusion criteria, and culturally specific content.

In addition to these contextual considerations, alternative explanations for the observed improvements warrant attention. Non-specific factors, including the Hawthorne effect, expectancy effects, and attention from study personnel, may have contributed to the observed improvements. While these factors cannot be entirely ruled out, several aspects of the study design reduce their plausibility as the sole or primary explanation. The inclusion of a control group that received comparable attention and assessment suggests that the observed group differences are unlikely to be attributable solely to attention. The blinding of outcome assessors to group allocation minimizes the risk of assessment bias. Additionally, the standardized training of facilitators and the use of a manualized protocol helped minimize variability in non-specific factors such as interpersonal style or enthusiasm. Nevertheless, the single-blind design and reliance on self-reported outcomes mean that these biases may not be fully eliminated, and the extent of their contribution remains uncertain. Future definitive trials should incorporate active control conditions matched for attention dose and time to more precisely isolate the effects of the intervention.

### 4.5 Clinical implications

The improvements in family resilience, coping style, family disease burden, and social support are likely to be perceptible in dyads’ daily lives. For patients, these improvements may translate into better emotional adjustment, reduced distress, and more effective management of treatment-related challenges. For caregivers, they may mean reduced role strain, increased confidence, and a greater sense of shared responsibility. Notably, the effect sizes in this study are comparable to or larger than those reported in similar dyadic interventions, and the maintenance of benefits at 3 months suggests durable improvements in dyadic functioning. The CFFRI offers structured professional guidance and continued follow-up to support the recovery process, helping dyads more effectively manage the multifaceted challenges of breast cancer. By integrating peer support and emphasizing dyadic coping, the intervention enhances access to social resources, reduces emotional distress, and strengthens confidence in disease management. Through its dyad-centered approach, the program also improves organizational and communication capacities within the family. Encouraging collaborative coping not only enhances immediate outcomes but also equips dyads to navigate illness-related crises and transitions more successfully, facilitating reintegration into daily life after treatment. As a systematically designed and feasibly delivered intervention, the CFFRI shows promise for clinical implementation and broader integration into psycho-oncology and supportive care services.

### 4.6 Study limitations

This study has several limitations that warrant consideration. First, the relatively small sample of 60 dyads was recruited from a single tertiary hospital in China, which limits the generalizability of the findings both within and beyond the Chinese cultural context. Patients in tertiary hospitals may differ from those in community or primary care settings in terms of disease severity, socioeconomic status, and health literacy, all of which may influence engagement and responsiveness. Future multi-center studies with larger and more diverse samples are needed to validate the efficacy of the CFFRI. Second, the intervention was delivered fully online due to pandemic-related restrictions. While this format enhanced accessibility and convenience, it may not be transferable to face-to-face settings, where different interaction dynamics and adherence patterns may emerge. Future comparative studies of online versus in-person delivery are needed to clarify the relative effectiveness and feasibility of different implementation modes and to support broader scalability. Third, the follow-up period was limited to 1 week and 3 months post-intervention, which precludes conclusions about the long-term sustainability of the observed benefits. Future studies should incorporate extended follow-up to evaluate the durability of intervention effects and to determine the optimal timing for potential booster sessions. Fourth, the study relied exclusively on self-reported outcomes from patient–caregiver dyads. Self-report measures are susceptible to social desirability bias and response bias, which may have overestimated intervention effects. Future studies should incorporate objective measures and reports from multiple family members to complement self-report data and minimize these biases. Fifth, as a pilot study, all findings should be interpreted as preliminary. The promising results require replication in larger, more diverse samples before definitive conclusions about the intervention’s efficacy can be drawn.

## 5 Conclusion

This pilot study provides preliminary evidence that the Coping-Focused Dyadic Family Resilience Intervention (CFFRI) is feasible and shows promise in enhancing family resilience and adaptive coping among breast cancer patient–caregiver dyads. The intervention appeared to reduce disease-related burden and strengthen relational capacities, helping dyads better recognize their strengths and navigate the illness experience more collaboratively. As a dyad-centered, strengths-based approach, the CFFRI represents a meaningful shift from conventional patient-focused models and may offer clinicians a practical tool for supporting families facing breast cancer. However, these findings are limited by the single-site design and modest sample size, and definitive conclusions await replication in larger, more diverse populations. Future research should employ multicenter trials, incorporate longer-term follow-up, and include multi-informant assessments to more fully evaluate the intervention’s impact within family systems.

## Data Availability

The raw data supporting the conclusions of this article will be made available by the authors, without undue reservation.
